# Relationship between Disease Resistance and Rice Oxalate Oxidases in Transgenic Rice

**DOI:** 10.1371/journal.pone.0078348

**Published:** 2013-10-24

**Authors:** Xian Yong Zhang, Zhuan Hua Nie, Wen Juan Wang, David W. M. Leung, Da Gao Xu, Bai Ling Chen, Zhe Chen, Lie Xian Zeng, E. E. Liu

**Affiliations:** 1 College of Life Science, South China Agricultural University, Guangzhou, China; 2 Plant Protection Institute, Guangdong Academy of Agricultural Sciences, Guangzhou, China; 3 School of Biological Sciences, University of Canterbury, Christchurch, New Zealand; 4 College of Natural Resources and Environment, South China Agricultural University, Guangzhou, China; Hokkaido University, Japan

## Abstract

Differential expression of rice oxalate oxidase genes (*OsOxO1-4*) in rice leaves (*Oryza sativa* L.) in response to biotic stress was assayed using RT-PCR. *OsOxO4* was induced transiently at 12 h in plants inoculated with the pathogens of bacterial blight and that of the wounding control. Inoculation with the rice blast pathogen induced *OsOxO2* expression compared to the mock spray control. Overexpressing *OsOxO1* or *OsOxO4* in rice resulted in elevated transcript levels of the respective transgene as well as *OsOxO3* in leaves compared to that in untransformed wild type (WT). In a line of RNA-i transgenic rice plants (i-12), expression of all four *OsOxO* genes except that of *OsOxO2* was severely inhibited. Oxalate oxidase (OxO, EC 1.2.3.4) activity in plants overexpressing *OsOxO1* or *OsOxO4* was substantially higher than that in WT and the RNA-i lines. It was found that transgenic rice plants with substantially higher OxO activity were not more resistant to rice blast and bacterial blight than WT. In contrast, some RNA-i lines with less OxO activity seemed to be more resistant to rice blast while some overexpressing lines were more susceptible to rice blast than WT. Therefore, OxO might not be a disease resistance factor in rice.

## Introduction

The first germin was found during a search for germination-specific proteins in wheat [1,2]. It has been identified as an oxalate oxidase (OxO, EC 1.2.3.4) which catalyzes the conversion of oxalate in the presence of O_2_ into H_2_O_2_ [3]. Proteins with 30 to 70% amino acid identities with germins were designated as germin-like proteins (GLPs) [4]. Most germins and GLPs occur as oligomeric glycoproteins and are located in the extracellular matrix [4,5]. Various studies have shown that germins and GLPs are associated with the response of plants under biotic stress. For example, the transcription of germin-like oxalate oxidase gene in wheat and barley leaves increased following pathogen attack [6,7], and OxO activity induced by powdery mildew fungus was found exclusively in the cell wall of barley leaf mesophyll cells. This has led to the hypothesis that OxO might be responsible for production of H_2_O_2_ which is involved in the regulation of the hypersensitive response during plant-pathogen interactions [6]. *OsOxO4* was induced after inoculation with *Magnaporthe oryzae* (*M. oryzae*) and *Xanthomonas oryzae* pv. *oryzae* (Xoo) [8], and some *OsGLPs* also could be induced by *M. oryzae* infection [9]. Expression of *BnGLP3* and *BnGLP12* in rape was up-regulated after *Sclerotinia sclerotiorum* infection, but up-regulation of *BnGLP12* expression only occurred in the disease-resistant line [10]. Overexpression of a wheat germin with OxO activity in soybean, rape and tomato led to enhanced resistance to S. sclerotiorum infection [11–13]. Addition of extracts containing OxO could completely inhibit sclerotia formation by S. sclerotiorum grown in potato dextrose broth [14]. Moreover, elevated levels of H_2_O_2_, salicylic acid and defence gene expression were observed in transgenic sunflower constitutively expressing a wheat *OxO* gene [15]. Therefore, OxO is thought to be involved in plant disease resistance [11,12,15]. However, the precise mechanism whereby OxO could contribute to plant disease resistance remains unclear.

Bioinformatics analysis showed that there are four *OxO* genes (Os03g0693700-Os03g0694000 and herewith referred to as *OsOxO1-4*) which are co-localized with a blast disease resistance QTL on chromosome 3 in the rice genome [16,17]. These genes share greater than 90% nucleotide identity, but their promoter regions are distinct, suggesting that the expression of these genes could be differentially regulated. Signal peptides of the four genes encoding polypeptides are predicted by SignalP (http://www.cbs.dtu.dk/services/SignalP/) to be present at the N-terminus, and OsOxOs are predicted to be secreted and localized in the extracellular matrix. Feng and Takano [18] reported that OxO might act as one of the downstream elements in the signal cascade of rice blast disease resistance mediated by an OSK_3_ protein kinase. Recently, the expression of *OsOxO4* gene was shown to be up-regulated earlier in rice blast resistant than in susceptible lines after inoculation with *M*. *oryzae*, suggesting that this gene might have important roles in resistance to rice blast [8]. In contrast, Kim et al. [19] showed that germin A (Os08g0189900) and *OsOxO3* (Os03g0693900) could also be induced by *M*. *oryzae*, but the levels of germin A and *OsOxO3* transcripts were both higher in compatible than in incompatible interactions at 48 h while the *OsOxO3* transcript level was higher in the incompatible interaction at 72 h.

Suppression in expression of *OsGLP* genes on chromosome 8 has been correlated with increased susceptibility to rice blast and sheath blight, suggesting that some of the 12 *OsGLP* genes located in the QTL region collectively conferred plant disease resistance [20]. Moreover, in transgenic rice plants down-regulating expression of *OsGLP1* (Os08g0460000 located on chromosome 8 which has no nucleotide identity with the 12 previously mentioned *OsGLP*s on the same chromosome) also resulted in increased susceptibility to sheath blight and rice blast [21]. Overexpressing OsGLP1 with inherent superoxide dismutase (SOD) activity in transgenic tobacco improved tolerance to *Fusarium solani*, and led to accumulation of more H_2_O_2_ and lignin in the vascular bundle of leaves compared to wild type [22]. Similarly, some members of GLPs, such as HvGER4d and HvGER5a [23], VvGLP3 [24], BnGLP3 and BnGLP12 [10] also exhibit SOD activity for the dismutation of superoxide into oxygen and H_2_O_2_ and have been shown to be associated with plant defence. Induction of OxO and SOD activities by S. sclerotiorum was, however, found in the susceptible *Phaseolus coccineus* variety and not in the more resistant one [25]. The sensitivity of the susceptible line of bean to oxalate toxicity and oxalate concentration in infected stem tissues ranked the highest. Since genetic differences in susceptibility to S. sclerotiorum among different P. coccineus lines are partially dependent on oxalic acid, OxO should not be considered as a resistance factor in the interaction between P. coccineus and S. sclerotiorum [25]. Therefore, the mechanism by which *OsGLPs* might influence plant defence is still elusive. 

Overall it seems that germins and GLPs are important for plant defence, but the precise mechanisms of their involvement remain to be elucidated further and only few of the specific gene family members have been studied in this regard. Here we investigated changes in expression of all four *OsOxO* gene family members and OxO activity under biotic stress. In addition, the tolerance of transgenic rice plants with altered *OsOxO* expression levels to *M. oryzae* and *Xoo* was evaluated. 

## Materials and Methods

### Plant material and culture conditions

Rice seeds (*Oryza sativa* L. subsp. *Japonica* Kato, Zhonghua11) were sterilized with 5% (v/v) NaOCl for 10 min followed by 3-5 rinses with tap water. The surface-disinfected seeds were soaked in deionized water for 12 h before they were germinated on a sheet of water-soaked filter paper in a Petri dish placed in a controlled growth chamber in the dark (28 °C). To study the effect of rice blast on *OsOxO* gene expression, the germinated seeds were transferred to soil and grown to the three-leaf stage in a glasshouse. Then fully expanded leaves were pooled after inoculation with *M*. *oryzae* or mocked-spray inoculation for 0, 6, 12, 24, 48 and 72 h. To study the effect of mechanical wounding and *Xoo* inoculation on *OsOxO* gene expression, the germinated seeds were pre-grown with complete Kimura B nutrient solution [26] in a glasshouse. When the seedlings reached the three-leaf stage, they were transferred to soil and grown to the booting stage, then leaves were harvested at 0, 12, 24 and 48 h after inoculation or mechanical wounding. 

### Extraction and assay of OxO activity

Freshly collected rice leaves or palea and lemma (about 10 mg) were ground in liquid nitrogen and processed for OxO activity determination according to the procedure of Zhang et al [27] with some modifications. The enzyme assay mixture contained 40 mM succinic acid/NaOH buffer at pH 3.8, 60 % (v/v) ethanol, 0.8 mM oxalic acid, 0.025% N, N-dimethylaniline, 0.1 mg/mL 4-aminoantipyrine and 5 units/mL of horseradish peroxidase. Trichloroacetic acid (TCA, 0.1%) was added after the mixture was incubated at room temperature for 5-60 min and then centrifuged at 12000×g for 5 min at 4 °C. The absorbance of the supernatant was measured at 555 nm against the control reaction without oxalic acid. OxO activity was determined as the amount of H_2_O_2_ (nmol) produced per min by enzyme extracts prepared from 1 g fresh tissue.

### Analysis of *OsOxO1-4* gene expression by semi-quantitative RT－PCR

RT-PCR was conducted to profile the expression patterns of four *OsOxO* genes. Total RNA was extracted using Trizol (Invitrogen, U.S.A), and then treated with DNaseI (Takara, Japan). About 1 μg RNA was used for cDNA synthesis in a 25 μL reaction volume with ReverTra Ace (Toyobo, Japan) and oligodT_(20)_ primer according to the manufacturer’s instructions. Amplification of the *actin* gene with an optimal number of PCR cycles in each sample was used as positive control for each *OsOxO* gene. PCR was performed using a PTC-200 machine (Bio-RAD, U.S.A), and the PCR products were separated on 1% (w/v) agarose gels and then visualized with Goldview (Amresco, U.S.A) staining. Primers used for RT-PCR experiments are listed in Table 1.

**Table 1:** Primer sets for RT-PCR analysis of *OsOxO1-4*.

Accession number	Gene	Forward primer(5'-3')	Reverse primer(5'-3')	size
Os03g0693700	*OsOxO1*	AAGAAATTG AGCCGAGACCTG	ACCACCCTGGAGTAGTACCTGTT	511
Os03g0693800	*OsOxO2*	ACCATA ACAGCTGGAGTGGTGTTCG	TGTCCACACGCAGCGCCTTAA	625
Os03g0693900	*OsOxO3*	TTCAAAGCAGCTGGGTTGGTGT	ACAATGTGAGCGGGACGAAGAC	568
Os03g0694000	*OsOxO4*	TTGTCACTGCGCTTCTTTCC	GCTCAACTACACCAGCATCCAC	766

### Culturing plant pathogens and inoculation methods

The conidium suspension of *M*. *oryzae* GDO8-T13 was obtained according to Yang et al [28]. Plants were spray inoculated with 1×10^5^ conidia per milliliter suspended in sterilized deionized water or sterilized deionized water alone (mock-spray inoculation). After inoculation, seedlings were put in a chamber in the dark at 100% relative humidity for 24 h before they were returned to the glasshouse. Cultures of *Xoo* SCX1-6 grown on slant peptone-sucrose agar (PSA) medium in the dark at 28 °C for 3 days were diluted to approximately 3×10^8^ cells per milliliter with sterile deionized water [29]. Rice leaves at the booting stage were inoculated by cutting off the leaf tip with scissors which had previously been dipped in the bacterial suspension or in sterile deionized water as the wounding control. Infection of the treated leaves was examined after 20 days of inoculation. 

### Generation of *OsOxO* silencing and overexpressing transgenic rice plants

A fragment of the conserved sequence of *OsOxOs* was amplified for use in silencing *OsOxO* genes. After digestion with restriction enzymes *Bam*HI and *Hin*dⅢ, the fragment was then ligated in RNAi vector pYLRNAi.5 (provided by Dr. Yaoguang Liu, South China Agricultural University) that contained two multi-cloning sites (MCS) separated by an intron. The fragment was firstly inserted in a sense orientation at MCS1 between *Bam*HI and *Hin*dⅢ. After cutting with the restriction enzymes and DNA sequencing confirmed the correct orientation and sequence of the fragment being 100% identical to the cDNA (Os03g0693700) reported in NCBI, a second fragment was amplified between the *Mlu*I and *Pst*I restriction sites at the ends from the ligated vector. This second fragment was then ligated at MCS2 between *Pst*I and *Mlu*I. In this way, an opposite orientation in contrast to the sequence at MCS1was generated. For overexpression of *OsOxO1* or *OsOxO4* in rice, fragments containing complete ORF sequences for *OsOxO1* and *OsOxO4* were cloned using RT-PCR. After digestion with restriction enzymes, the resulting fragments were cloned in the transformation vector pOX (provided by Dr. Yaoguang Liu, South China Agricultural University) containing the hygromycin resistance gene as a selectable marker under the control of ubi as a promoter. After cutting with restriction enzymes and DNA sequencing confirmed the correct orientation and the sequences of the fragments being 100% identical to the respective cDNAs (Os03g0693700 and Os03g0694000) reported in NCBI, the foresaid vectors were then transformed into rice callus via an *Agrobacterium*-mediated transformation procedure according to Hiei et al [30] with some modifications. Transgenic rice plants that showed a single T-DNA insertion in T0 and 3:1 segregation ratios in the T1 were bred to obtain homozygous lines which were screened for changes in *OsOxOs* mRNA levels by RT-PCR analysis and further characterization. 

### Transgenic rice lines in response to biotic stress

Transgenic rice seedlings were grown in soil to the three-leaf stage in a glasshouse and then inoculated with *M*. *oryzae* GDO8-T13. Infection was examined at 7 d after inoculation. When seedlings were grown to the booting stage, leaves from transgenic lines and wild-type plants were inoculated with *Xoo*. At the preliminary stage of head sprouting, leaf sheaths were unwrapped and the enveloped young ear panicles were inoculated with *M*. *oryzae* GDO8-T13. Infection was examined after 20 days of inoculation.

### Localization of OxO activity in rice roots

Root tissue samples were frozen and cut using a freezing microtome into 30 µm thick cross sections which were transferred to glass slides and immersed in a developing solution containing 40 mM succinic acid/NaOH buffer at pH 3.8, 60 % (v/v) ethanol, 2 mM oxalic acid, 0.5 mg/L 4-chloro-1-naphthol and 5 units/mL of horseradish peroxidase. After about 5 min, the staining patterns of the sections were photographed under a light microscope (Leica, German).

### Statistical analysis

For each treatment, data were statistically analyzed using MS Excel for Windows, and significant differences between various treatments were analyzed using the Duncan's new multiple range method of the DPS v6.55 (DPS Soft Inc., Tang, Hangzhou, China.) analytical software.

## Results

### Effects of bacterial blight and rice blast on expression of *OsOxO* genes in rice leaves

**Figure 1:**
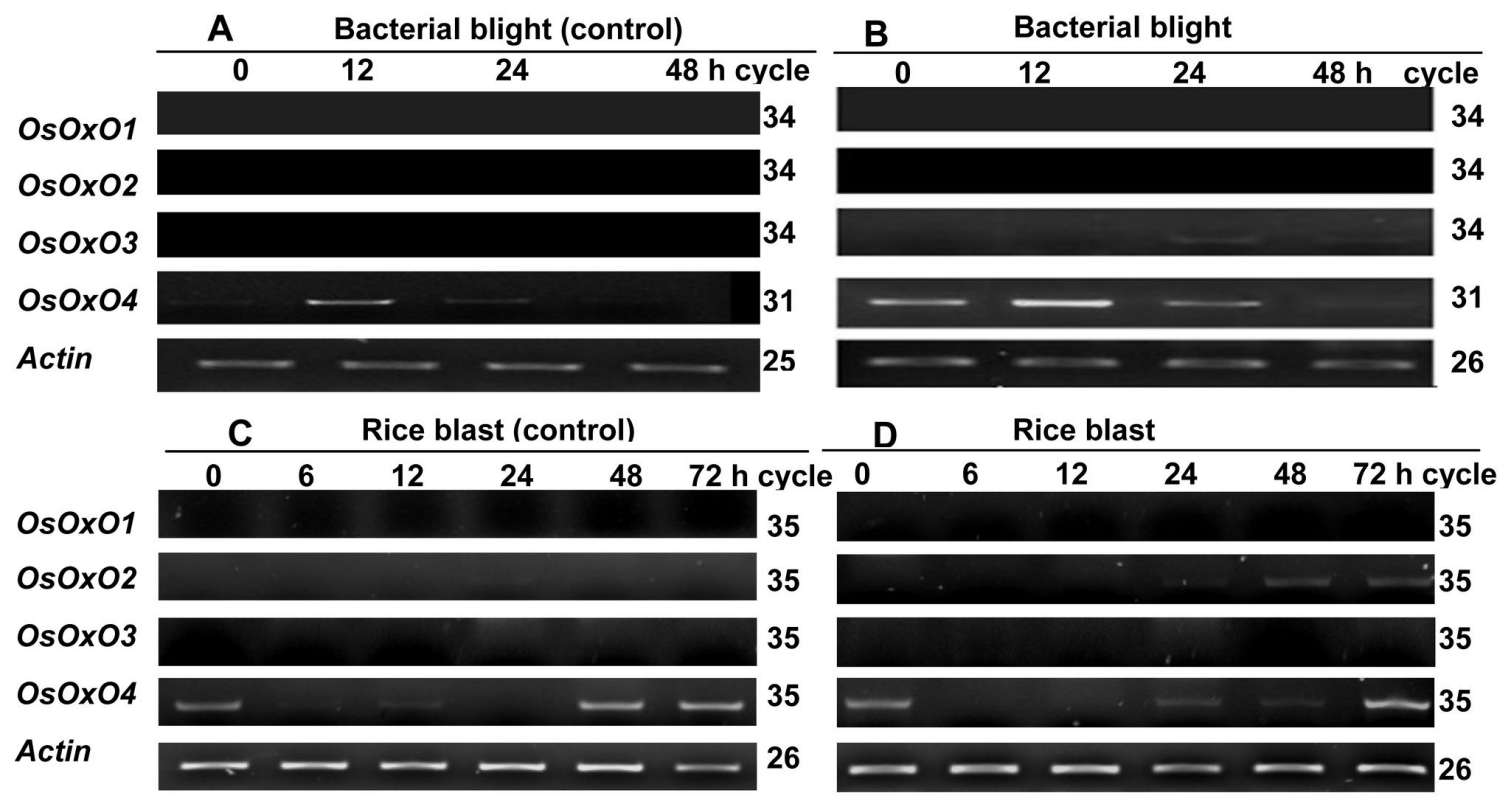
Expression of *OsOxO1-4* in rice leaves in response to pathogen inoculation. When Zhonghua 11 seedlings were grown to the booting stage, leaves were wounded without *Xoo* SCX1-6 cultures (mock inoculation control) (A) and inoculated with *Xoo* SCX1-6 (B). Three-leaf stage Zhonghua 11 seedlings were spray inoculated with water (C) and *M*. *oryzae* GDO8-T13 (D). Then total RNA was extracted from the treated leaves at 0, 12, 24 and 48 h after inoculation for analysis using semi-quantitative RT-PCR.

Only *OsOxO4* transcript was detected in leaves at time 0 (before respective pathogen inoculation and respective controls). Differential expression of the four *OsOxO* gene family members was found in rice leaves following inoculation with the respective pathogen causing bacterial blight, or rice blast and the respective mock inoculation controls (Figure 1). The transcripts of *OsOxO1-3* were not detected or at very low levels in rice plants inoculated with *Xoo* and that of the mock inoculation (wounding control) (Figure 1 A and B). In contrast, expression of *OsOxO4* was induced in response to inoculation with *Xoo* and wounding (mock inoculation control) at 12 h (Figure 1 A and B). However, by 24 h the *OsOxO4* transcript decreased to levels similar to those at earlier times in the mock inoculation controls and the plants inoculated with *Xoo* (Figure 1 A and B). *OsOxO1* and *OsOxO3* transcripts were not detected in plants inoculated with *M. oryzae* and those of mock spray inoculation (Figure 1 C and D). The *OsOxO2* transcript exhibited a higher level in plants inoculated with *M. oryzae* at 48 and 72 h but was not induced in those of the mock spray control. Similar expression patterns of *OsOxO4* were found in plants inoculated with *M. oryzae* and those of the mock spray control. 

### Characterization of transgenic plants

**Figure 2:**
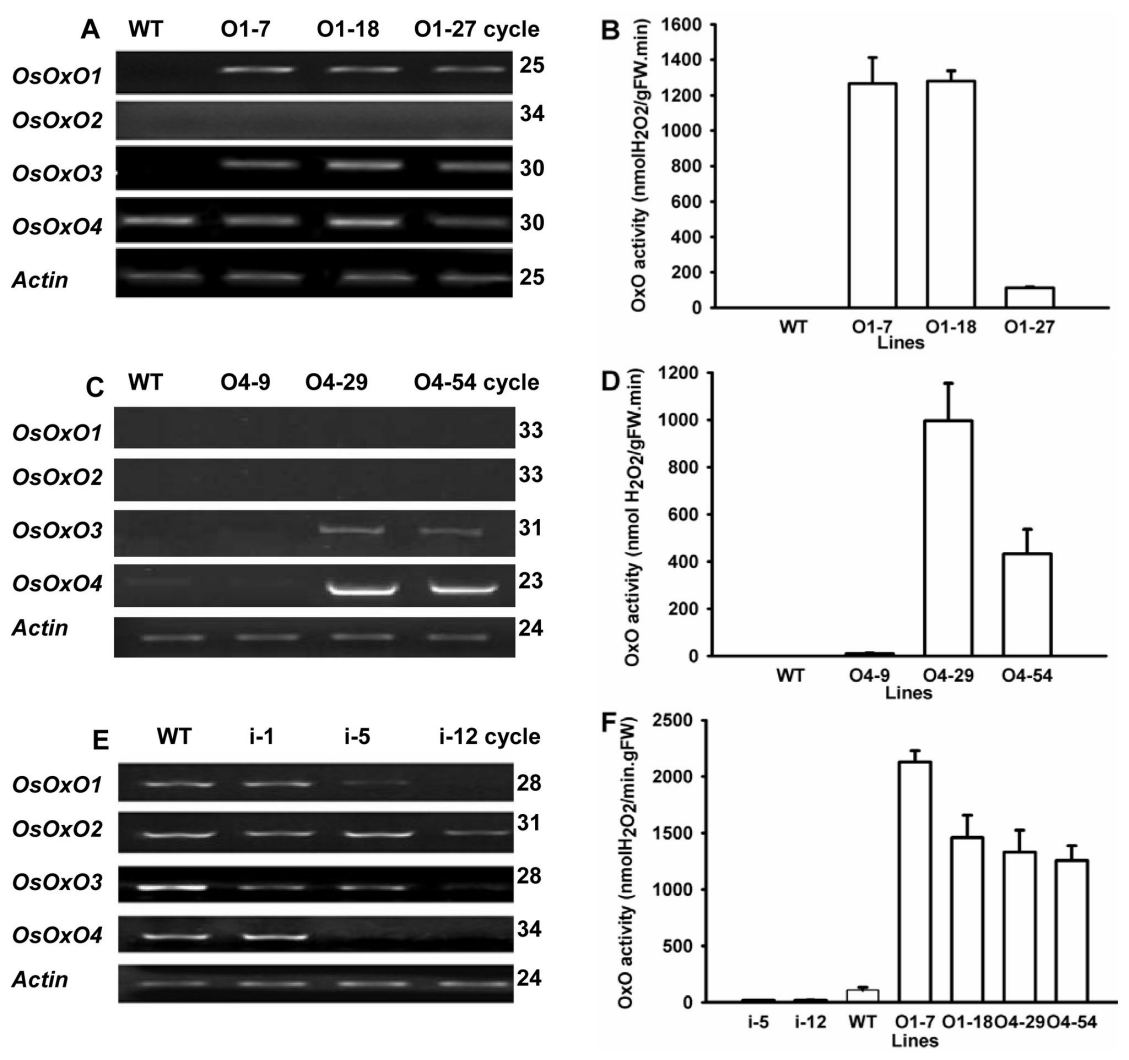
Levels of *OsOxO1-4* transcripts and OxO activity of transgenic rice lines. Levels of *OsOxO1-4* transcripts (A, C) and OxO activity (B, D) in leaf tissues at the three-leaf stage of transgenic rice lines overexpressing *OsOxO1* (O1-7, O1-18 and O1-27), *OsOxO4* (O4-9, O4-29 and O4-54) and wild type (WT). Levels of *OsOxO1-4* transcripts (E) in palea and lemma from *OsOxOs-*RNAi transgenic lines (i-1, i-5 and i-12) and wild type (WT) at 20 d after anthesis. OxO activity (F) in palea and lemma from *OsOxOs-*RNAi transgenic lines (i-5 and i-12), wild type (WT) and overexpressing transgenic lines (O1-7, O1-18, O4-29 and O4-54) at 15 d after anthesis.

To determine whether expression of *OsOxO* can confer resistance against several rice pathogens, transgenic rice plants constitutively overexpressing *OsOxO1* or *OsOxO4* or silencing *OsOxOs* were generated successfully. 60 and 54 lines overexpressing *OsOxO1* and *OsOxO4*, respectively, and 22 silencing lines were obtained. Southern blotting of genomic DNA showed integration of a single copy of the T-DNA (data not shown) into the genomes of several overexpressing lines (O1-7, O1-18, O1-27, 04-9, 04-29 and 04-54), and silencing lines (i-1, i-5, and i-12) which were chosen for further characterization. The transgenic plants exhibited normal growth similar to that of the wild type (Figure S1). At the three-leaf stage, expression of *OsOxO1* and *OsOxO3* was detectable in the leaves of 01-7, 01-18 and 01-27 but not in those of WT (Figure 2A). Expression of *OsOxO2* was not affected by overexpression of *OsOxO1* as the transcript of *OsOxO2* was not detectable in the leaves of the three transgenic lines as well as in those of WT (Figure 2A). The OxO activities in the leaves of 01-7 and 01-18 were substantially higher than that in the leaves of WT while that in 01-27 was several folds lower than the other two overexpressing lines but still higher than that in WT (Figure 2B). Overexpression of *OsOxO4* had no effect on the expression of *OsOxO1* and *OsOxO2* as their transcripts were not detectable in the leaves of 04-9, 04-29 and 04-54 as well as in those of WT (Figure 2C). The expression of *OsOxO3* and *OsOxO4* was also not affected in 04-9 but was strongly elevated in 04-29 and 04-54 (Figure 2C). This correlated with the lowest level of OxO activity found in the leaves of 04-9 which was similar to that in WT (Figure 2D). The levels of OxO activity found in the leaves of the three overexpressing lines were in the following decreasing order: 04-29, 04-54 and 04-9. In the palea and lemma of i-1, the transcript levels of *OsOxO1-4* were apparently the same as those of WT (Figure 2E). In i-5, the expression of *OsOxO1* and *OsOxO4* was inhibited more strongly than that of the other two gene family members and expression of all four *OsOxO* genes but that of *OsOxO2* was severely inhibited in i-12. It was also confirmed that the OxO activities in the palea and lemma of both i-5 and i-12 were greatly reduced in comparison to that in WT (Figure 2F). By contrast, the levels of OxO activity in the palea and lemma of four overexpressing lines (O1-7, O1-18, O4-29, and O4-54) were substantially higher than those in WT and the silencing lines. In each of the four overexpressing lines, the OxO activities in the palea and lemma were apparently higher than those in the leaves (compare Figure 2 B, D and F).

**Figure 3:**
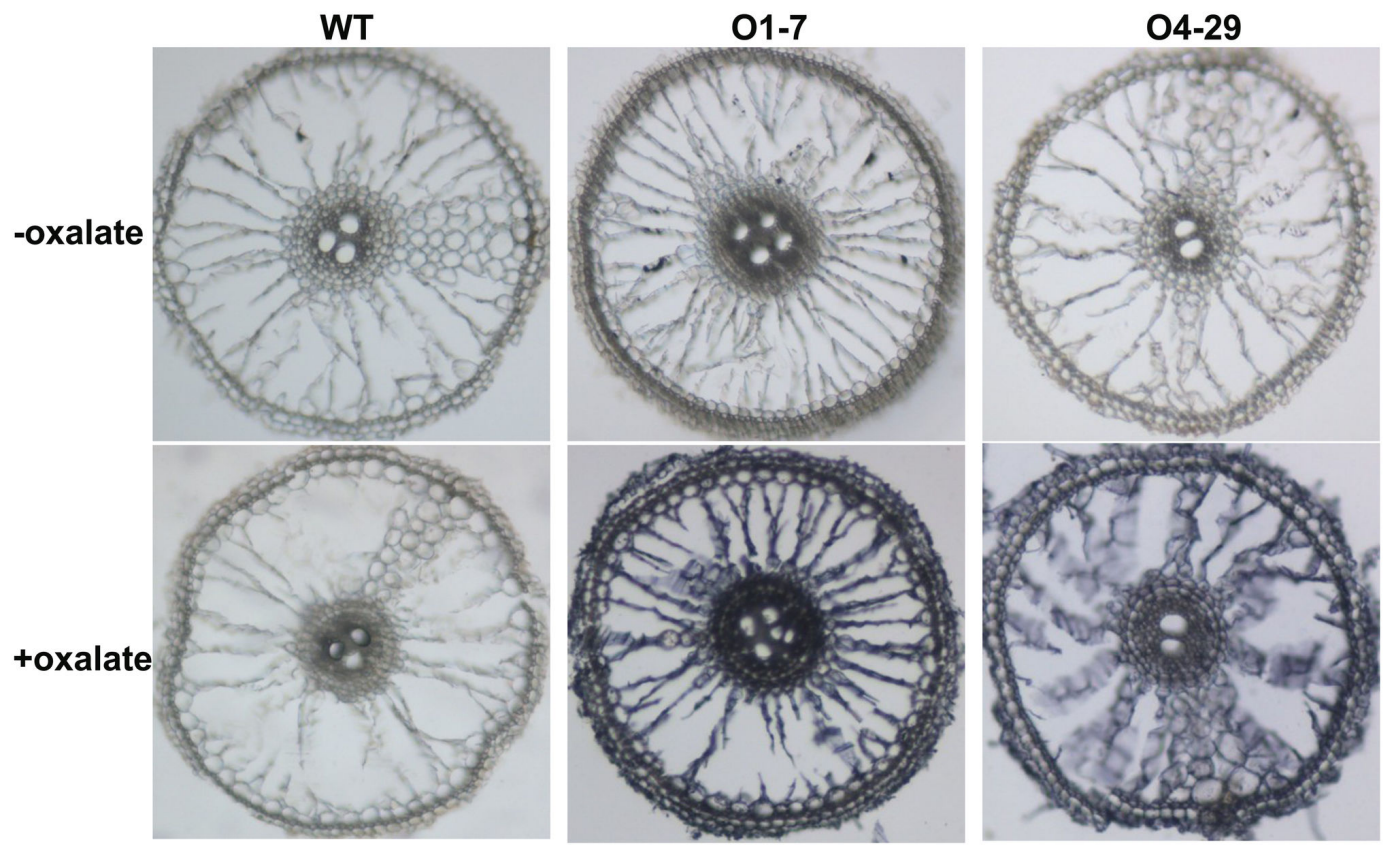
Staining of OxO activity in transverse root sections of transgenic rice plants and wild-type.

 Most OxO activity remained in the pellet after centrifugation of extracts from OxO overexpressing transgenic plants. Activity staining in cross sections of roots (Figure 3) revealed that the OxO activity in both *OsOxO1* and *OsOxO4* overexpressing lines was located mainly in the extracellular matrix (cell wall). 

Response of transgenic rice lines to inoculation with rice blast and bacterial blight pathogens

**Figure 4:**
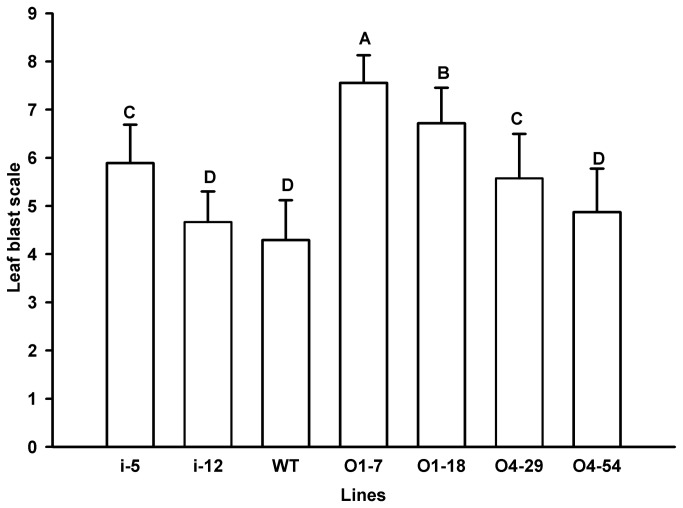
Scale of rice blast in leaves of transgenic lines and wild-type rice plants. Transgenic lines O1-7, O1-18, O4-29, O4-54, i-5 and i-12 and wild type (WT) at the three-leaf stage were inoculated with *M*. *oryzae* GDO8-T13. Leaf blast symptom was examined at 7 d after inoculation.

**Figure 5:**
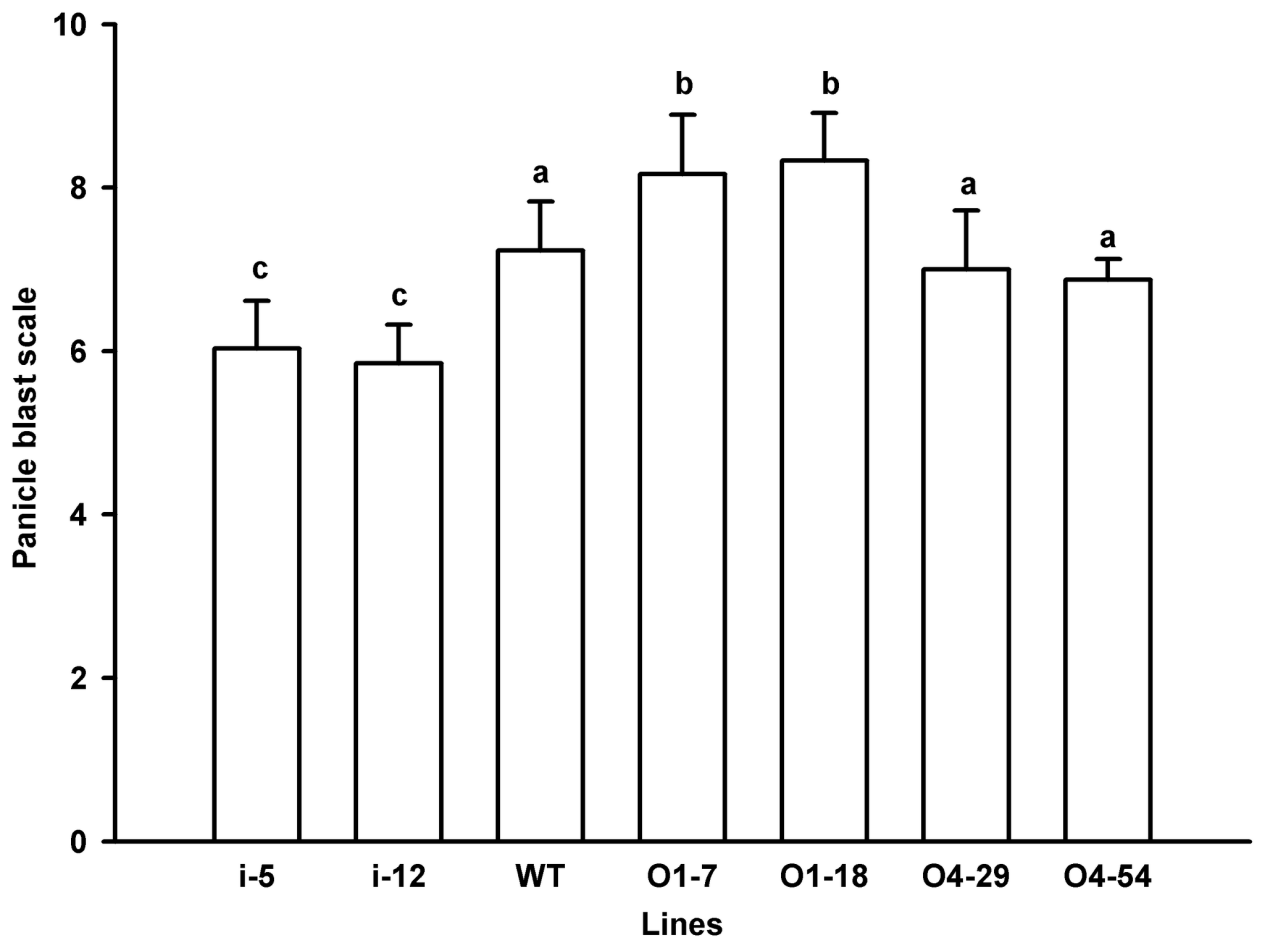
Panicle blast scale of transgenic rice lines and wild-type. At the preliminary stage of head sprouting, panicles from transgenic lines O1-7, O1-18, O4-29, O4-54, i-5 and i-12 and wild type (WT) were inoculated with *M*. *oryzae* GDO8-T13. Panicle blast scale was examined after 20 days of inoculation.

**Figure 6:**
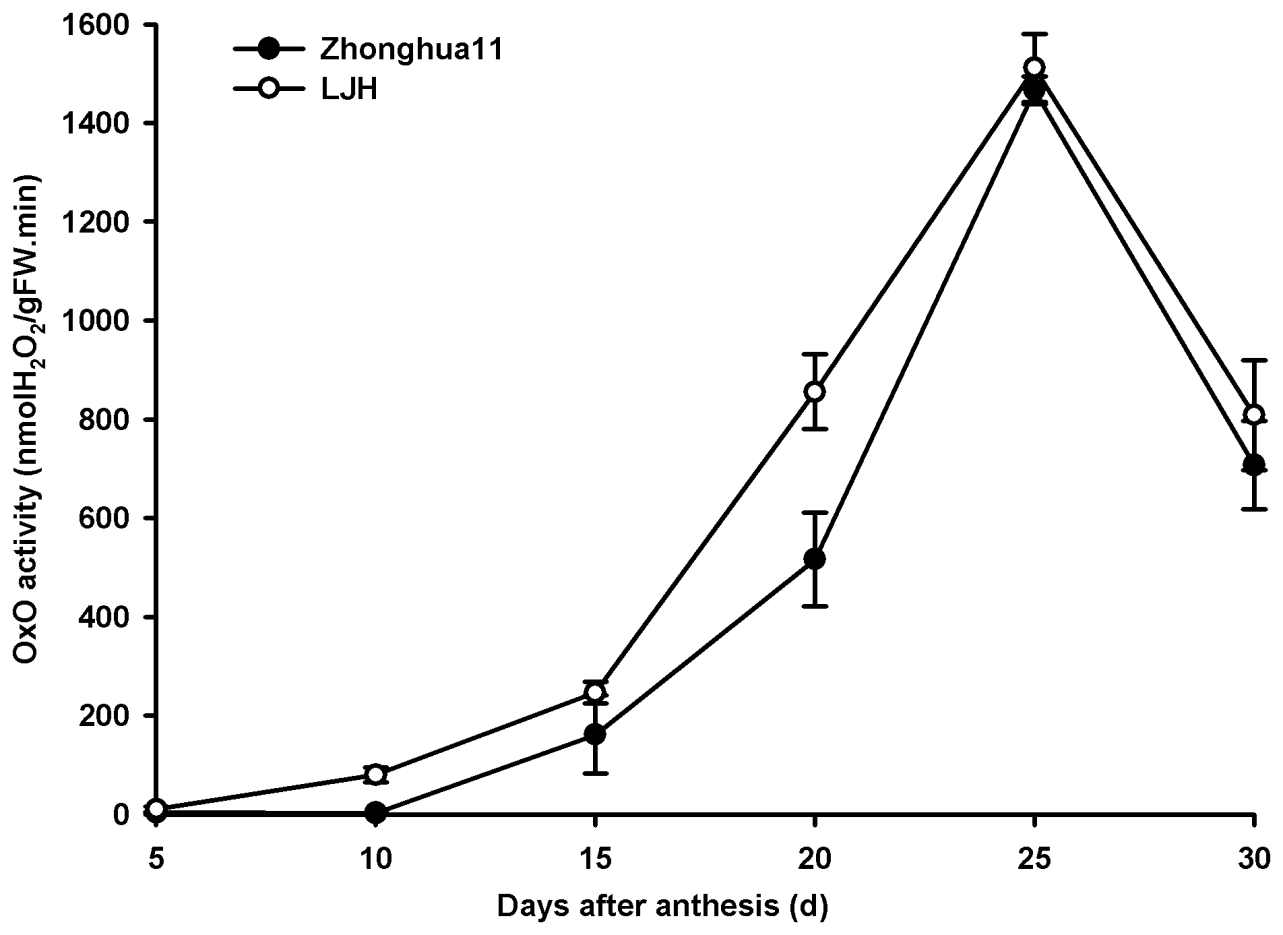
OxO activity in palea and lemma during rice seed development. Palea and lemma were sampled for OxO activity at 5, 10, 15, 20, 25 and 30 d after anthesis of Lijiangxintuanheigu (LJH) and Zhonghua 11 wild type.

**Figure 7:**
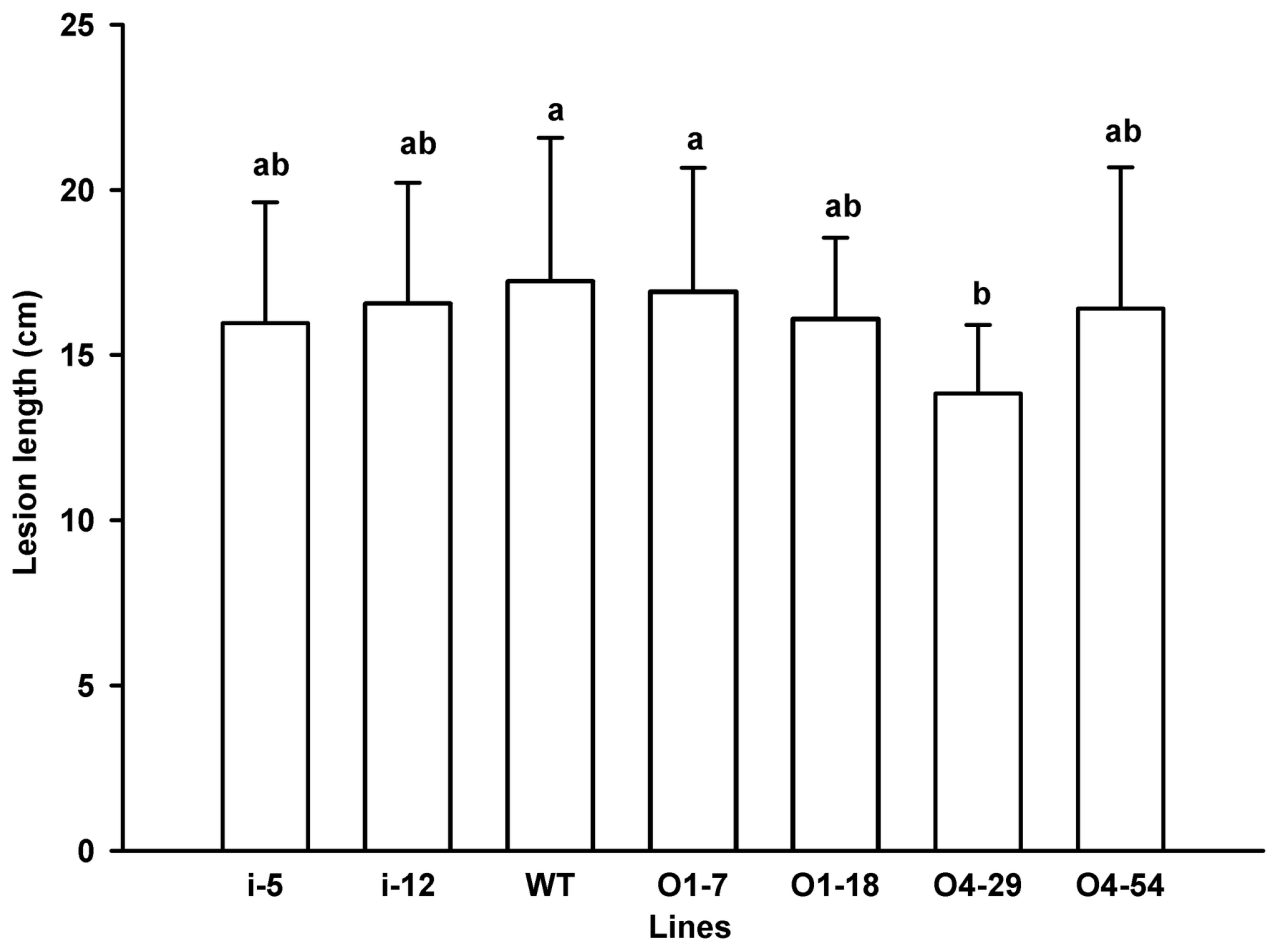
Lesion lengths in leaves of transgenic rice lines. Leaves at the booting stage of rice transgenic lines O1-7, O1-18, O4-29, O4-54, i-5 and i-12 and wild type (WT) were inoculated with *Xoo* SCX1-6. Lesion lengths of inoculated leaves were determined after 20 days of inoculation.

No obvious difference was observed in disease resistance to rice blast among WT, O4-54 and i-12, while O4-29 and i-5 exhibited a slightly higher level of disease severity than WT (Figure S2, Figure 4). By contrast, the susceptibility to rice blast of O1-7 and O1-18 increased remarkably compared to WT (Figure 4). The response of the transgenic rice lines to panicle blast was also investigated. There was no significant difference among WT, O4-29 and O4-54 in response to inoculation of the panicles with *M. oryzae*, while WT exhibited a lower panicle blast scale than O1-7 and O1-18 but a higher panicle blast scale than that of i-5 and i-12 (Figure 5). Interestingly, OxO activity in the palea and lemma of WT was 106.93 nmol H_2_O_2_/gFW. min which was remarkably (at least 10-fold) lower than that in O1-7, O1-18, O4-29 and O4-54 but higher than that in i-5 and i-12 (Figure 2F). Moreover, the trend and the levels of OxO activity in the palea and lemma of *M. oryzae* susceptible variety LJH were similar to those in the wild-type Zhonghua 11 during seed development (Figure 6). There was no significant difference in resistance of several *OsOxOs* overexpressing or RNA-i lines except 04-29 to bacterial blight (Figure 7) compared with WT.

## Discussion

The members of the *OsOxO* gene family exhibited different temporal and spatial expression patterns. *OsOxO1* was mainly expressed in the palea and lemma while *OsOxO2-4* mainly in rice seedlings. The transcript level of each gene was regulated developmentally (unpublished). A survey of various EST libraries found *OsOxO4* ESTs not only in those of healthy rice root, shoot and leaf, but also in those of plants under drought, cold and metal (CuSO_4_) stress. The expression of *OsOxO4* was increased after inoculation with *M*. *oryzae* and *Xoo*, insect or mechanical damage [8]. Moreover, many *OsGLP* of the GER4 subfamily and one GER3 (OsGLP8-12) were induced by *M*. *oryzae*, while most of the *OsGLP* genes were also induced by mock inoculations [9]. However, our results showed that there was no stimulation in expression of *OsOxO4* as a result of inoculation with *M*. *oryzae*, and *OsOxO2* was induced, elevated expression of *OsOxO4* at 12 h after wounding and inoculation with *Xoo*, suggesting that expression of this gene might not be associated with bacterial blight. The increases of *OsOxO* transcripts maybe due to an increase in hydrogen peroxide (H_2_O_2_) in rice seedlings because H_2_O_2_ could be produced in response to a variety of stimuli including wounding (mock-inoculation) and pathogen infection. It has been shown that there was an increased accumulation of H_2_O_2_ in leaf tissues of a resistant rice variety after inoculation with *M. oryzae* [9]. The transcripts of *HvGER1, HvGER4, HvGER5* and *NaGLP* were induced by exogenous application of H_2_O_2_ [23,31]. Moreover, our results showed that the levels of *OsOxO1-4* transcripts all increased in rice roots and leaves following H_2_O_2_ treatment (unpublished) and would seem therefore be consistent with the notion that differences in *OsOxOs* expression patterns in rice plants under a variety of stresses could be due to differential accumulation of H_2_O_2_ in response to stress.

 Many *GLPs* were associated with QTLs for disease resistance. For example, *OsOxO1-4* were co-localized with a blast disease resistance QTL on chromosomes 3 in the rice genome [17], and *HvOXOLP* was co-localized to a wheat QTL for resistance against *Pyrenophora tritici-repentis* [32]. Overexpression of a wheat or barley *OxO* gene enhanced the resistance of plants to diseases [11,12,15,33]. Down-regulation of the SOD-active *OsGLP1*（Os08g0460000 on chromosome 8 in the rice genome) made the transgenic rice plants more susceptible to sheath blight and rice blast [21], while overexpressing *OsGLP1* in tobacco improved the tolerance to *Fusarium solani* [22]. When *OsGLP* genes were silenced, the plants became more susceptible to *M*. *oryzae* and *Rhizoctonia solani* [20]. Nevertheless, it is not known if the proteins encoded by the genes in the previous studies possessed SOD or OxO activity. Therefore, it is possible that the decrease in resistance to rice blast exhibited by transgenic rice with down-regulated *OsGLP* expression might have nothing to do with OxO activity. On the contrary, our results showed transgenic rice lines overexpressing *OsOxO1* or *OsOxO4* did not show improved resistance to rice blast. Furthermore, the transgenic rice lines overexpressing *OsOxO1* were more susceptible to rice blast than WT and *OsOxOs*-RNAi transgenic rice lines were more resistant to panicle blast than WT, in spite of a higher level of OxO activity in WT than the silencing lines. Moreover, OxO activity in the palea and lemma of the more susceptible rice variety LJH was slightly higher than that in Zhonghua 11 (or WT). The results indicated rice blast resistance was not correlated with variation in OxO activity in different lines of rice plants. Interestingly, transient silencing of *HvGER3* made barley more resistant to *Blumeria graminis* [23]. Moreover, induction of OxO by S. sclerotiorum only occurred in a susceptible P. coccineus variety which had a higher oxalate level and was more susceptible to oxalate toxicity compared to a resistant variety, indicating OxO should not be considered as a disease resistance factor [25]. The expression level of *OsGLP8-12* (Os08g0231400) was higher in a susceptible variety than in a resistant variety, but that expression level of *OsGLP8-6* (Os08g0189500) was higher in the resistant variety [9]. Although some reports have shown that OxO had important roles in disease resistance, the pathogenic factor of most diseases caused by S. sclerotiorum was oxalate [11,12,15]. In these previous studies, the disease resistance mechanism of the transgenic plants overexpressing OxO was mainly due to the increased OxO activity being able to catalyze the degradation of the S. sclerotiorum toxin oxalate and producing the defense-inducing molecule H_2_O_2_ which has been demonstrated to play important roles in combating various diseases in plants. 

Interestingly, overexpression of *OsOxO1* enhanced expression of *OsOxO3* but reduced *OsOxO4* expression in some of the transgenic rice lines. Similarly, overexpression of *OsOxO4* enhanced the expression of *OsOxO3* but had no effect on expression of the other two gene family members. The mechanism underlying the observed gene interactions is not clear. This, however, suggests that in the transgenic rice lines overexpressing *OsOxO1* or *OsOxO4* and the RNA-i lines, activation or suppression of other defence-related genes might occur. This could influence the observed performance of these transgenic lines grown under biotic stress in this study.

In conclusion, the responses of the transgenic rice lines to biotic stresses indicated that overexpression of *OsOxO1* or *OsOxO4* cannot improve resistance to rice blast and bacterial blight, and that *OsOxO1* might make rice more susceptible to rice blast. It is doubtful that OxO is a disease resistance factor in rice.

## Supporting Information

Figure S1 **Appearance of transgenic and wild-type rice seedlings before inoculation with *M*. *oryzae* GDO8-T13.**
(TIF)

Figure S2 **Appearance of leaves from transgenic and wild-type rice plants at 7 d after inoculation with *M*. *oryzae* GDO8-T13.**
(TIF)
