# Supplementary figures and images for: Relationship between Disease Resistance and Rice Oxalate Oxidases in Transgenic Rice
Source: PLoS One. 2013 Oct 24;8(10):e78348. doi: 10.1371/journal.pone.0078348 (PMC3813443; doi:10.1371/journal.pone.0078348)

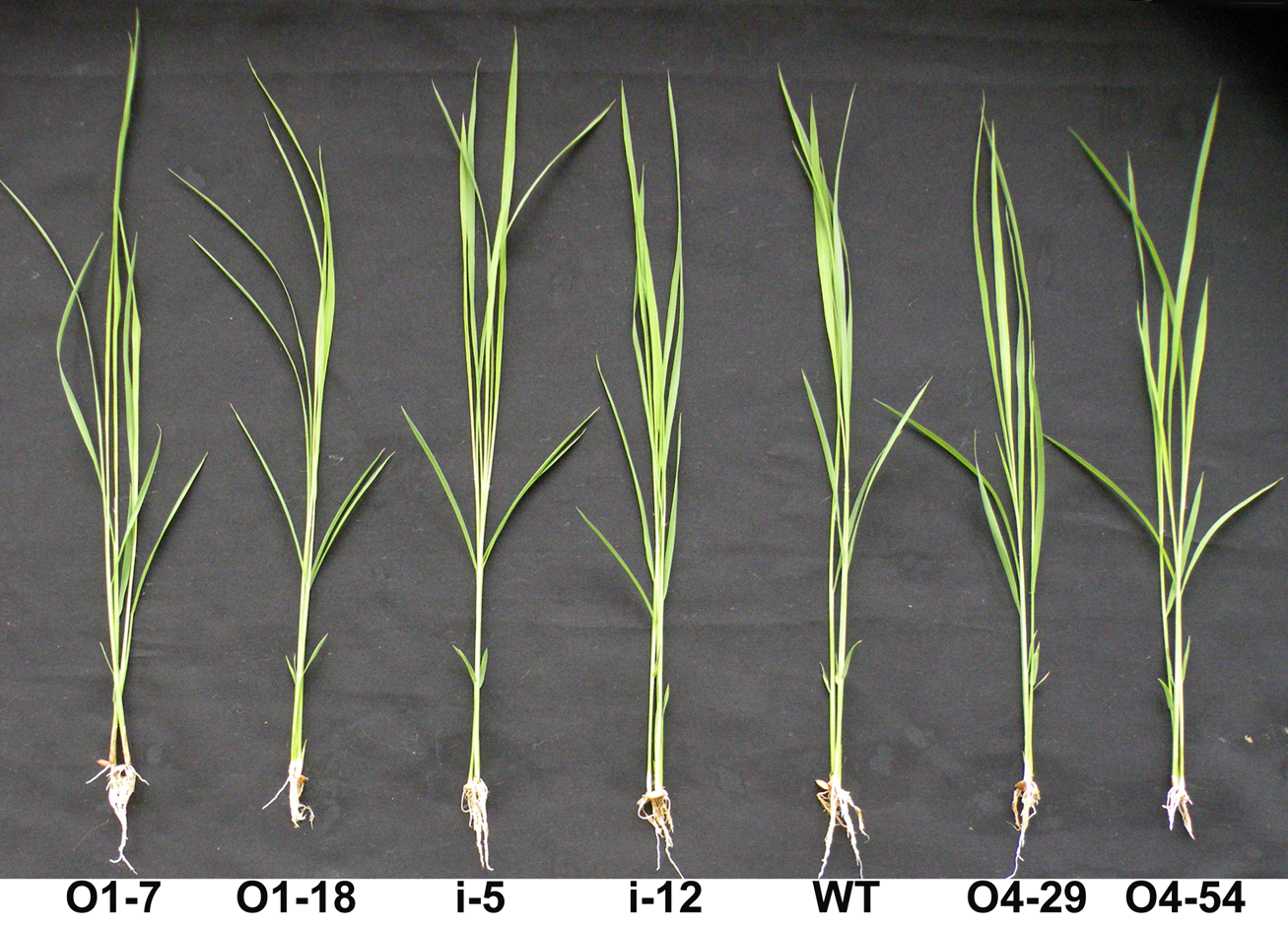

Supplement: Figure S1 — Appearance of transgenic and wild-type rice seedlings before inoculation with M. oryzae GDO8-T13. (TIF) [file pone.0078348.s001.tif]

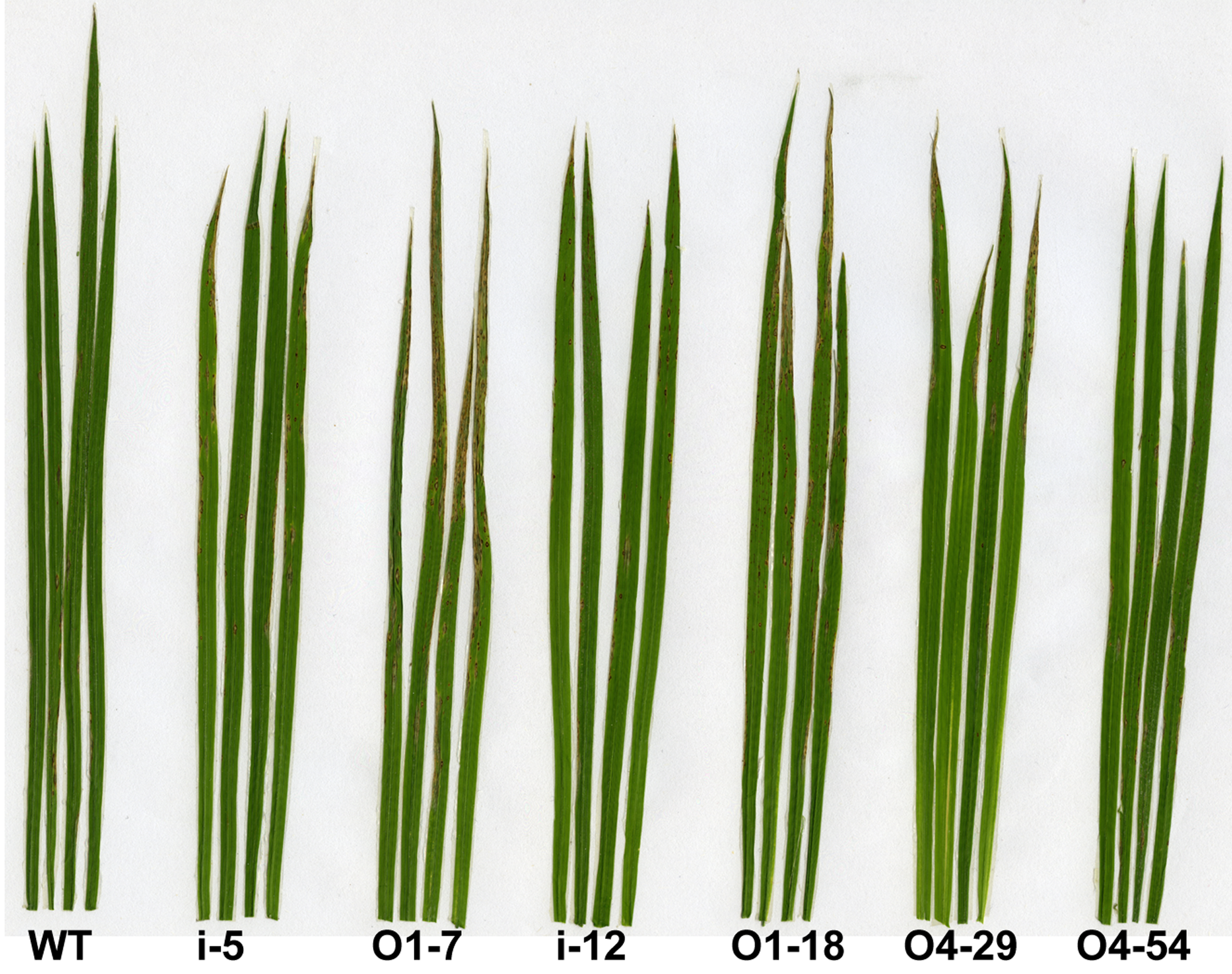

Supplement: Figure S2 — Appearance of leaves from transgenic and wild-type rice plants at 7 d after inoculation with M. oryzae GDO8-T13. (TIF) [file pone.0078348.s002.tif]
